# Magnolol inhibits porcine epidemic diarrhea virus infection by suppressing cathepsin L expression *in vitro* and *in vivo*

**DOI:** 10.1128/jvi.00137-26

**Published:** 2026-06-25

**Authors:** Yi-fan Liang, Xi Li, Gao-xi Zhu, Yi Song, Xiong-nan Chen, Heng Wang, Gui-hong Zhang

**Affiliations:** 1 Guangdong Provincial Key Laboratory of Zoonosis Prevention and Control, College of Veterinary Medicine, South China Agricultural University 554665, Guangzhou, China; 2 Key Laboratory of Animal Vaccine Development, Ministry of Agriculture and Rural Affairs 714755, Beijing, China; University of North Carolina at Chapel Hill, Chapel Hill, North Carolina, USA

**Keywords:** CTSL, magnolol, PEDV, antiviral activity, coronavirus infection, intestinal epithelial cells

## Abstract

Porcine epidemic diarrhea (PED), caused by the porcine epidemic diarrhea virus (PEDV), is an acute and highly contagious intestinal disease in pigs. The high variability of the PEDV S gene has rendered current commercial vaccines ineffective, indicating the need for safe, effective, and cost-effective antiviral agents to control PEDV. Here, we aimed to screen and identify compounds with anti-PEDV activity and elucidate the antiviral mechanisms of magnolol. A compound library was screened using an in-cell Western blotting assay to identify potential antiviral agents. The *in vitro* antiviral activity of magnolol was evaluated using viral titer assays, time-of-addition experiments, and Western blotting. The *in vivo* efficacy of magnolol against PEDV was assessed by monitoring body weight, rectal temperature, histopathological changes, and diarrhea scores. Cathepsin L (CTSL) expression and distribution were analyzed *in vitro* and *in vivo* using RT-qPCR, Western blotting, and confocal immunofluorescence. A total of 25 potential antiviral candidates were identified, among which magnolol exhibited significant anti-PEDV activity. Magnolol effectively inhibited the entry of PEDV, porcine deltacoronavirus, and porcine enteric alphacoronavirus by suppressing CTSL expression. *In vivo* experiments showed that magnolol treatment alleviated PED-associated pathological damage and altered CTSL distribution in the intestinal epithelium of piglets, thereby reducing PEDV infection. Mechanistically, magnolol inhibited PEDV infection by downregulating CTSL expression and altering its distribution in intestinal epithelial cells. This study provides valuable experimental evidence and suggests potential therapeutic strategies for the clinical treatment of porcine enteric coronavirus infections.

**IMPORTANCE:**

Porcine epidemic diarrhea (PED) is an acute and highly contagious intestinal disease in pigs that causes severe intestinal damage and death, especially in piglets. Currently available treatments and vaccines for the disease have been unable to provide complete protection due to the high mutation/variability of the causative virus, porcine epidemic diarrhea virus (PEDV). In this study, we demonstrated for the first time that magnolol, an extract from the roots and bark of *Magnolia officinalis*, inhibits PEDV infection by downregulating cathepsin L expression and altering its distribution in intestinal epithelial cells, thereby alleviating PED-associated pathological damage in the intestines of piglets. Our findings suggest that magnolol has potential applications in the treatment of PED.

## INTRODUCTION

Porcine epidemic diarrhea virus (PEDV) was first identified in the United Kingdom in 1971 (1). PEDV is an enveloped virus with a positive-sense, single-stranded RNA genome of 28 kb, which contains a 5′ untranslated region (UTR), a 3′ UTR, and at least seven open reading frames (ORF1a, ORF1b, and ORF2–6) responsible for encoding four structural proteins (spike [S], envelope [E], membrane [M], and nucleocapsid [N]) and three non-structural proteins (replicase polyprotein 1a and 1ab and ORF3) (2). PEDV infection causes vomiting, diarrhea, dehydration, and weight loss in pigs, with mortality rates reaching 100% in neonatal piglets. Importantly, the virus primarily targets the jejunum and ileum, with the duodenum being affected to a lesser extent. Infected intestinal villous epithelial cells undergo acute necrosis and detach from the lamina propria, leading to marked villous atrophy in the small intestine (3). In 2010, a large-scale PEDV outbreak occurred in China, leading to massive piglet mortality, with a 100% mortality rate in neonatal piglets (4). PEDV epidemics continue to cause substantial economic losses in swine herds worldwide, particularly during cold winters and springs. Although multiple inactivated and live-attenuated vaccines targeting both classical and variant PEDV strains are commercially available, they fail to provide effective protection.

Cathepsin L (CTSL) is a cysteine protease belonging to the papain superfamily that is predominantly localized in endosomes and lysosomes, where it is activated under acidic conditions (5). Research findings indicate that cathepsins are involved in viral entry. For instance, CTSL and CTSB cleave the GP1 protein of the Ebola virus, thereby facilitating infection (6). Additionally, reoviruses require cathepsin activation to induce membrane fusion (7). In SARS-CoV, CTSL enhances viral entry by mediating the proteolytic processing of the S1 subunit of the spike protein (8). Similarly, CTSL and CTSB can cleave the PEDV spike protein into the S2 subunit, thereby activating viral entry into host cells (9). Overall, these findings highlight the pivotal role of CTSL in coronavirus infections and suggest that it is a promising target for antiviral therapy against PEDV infection.

Magnolol is a lignan extracted from the roots and bark of *Magnolia officinalis*, which has been used in traditional Chinese medicine for thousands of years to treat anxiety, asthma, gastrointestinal disorders, headaches, and allergies (10, 11). In animal production, magnolol has been used as a feed additive to enhance growth performance, maintain intestinal health, and improve mucosal barrier integrity (12, 13). Magnolol exhibits several pharmacological activities, including antidepressant, anticancer, antioxidant, and autophagy-modulating effects (14–16). Recent studies have demonstrated the antiviral potential of magnolol. Chen et al. demonstrated that magnolol inhibits grass carp reovirus (GCRV) infection and suppresses GCRV-induced apoptosis (17). Moreover, magnolol, an active compound in the traditional Chinese medicine Huashi Baidu decoction, demonstrated dose-dependent inhibitory effects against SARS-CoV-2 infection (18). Furthermore, magnolol suppresses infections caused by enterovirus 71, influenza A virus, porcine reproductive and respiratory syndrome virus, norovirus, and hepatitis B virus (19–23). Collectively, these findings suggest that magnolol possesses broad-spectrum antiviral properties and is a promising candidate for the development of novel antiviral agents.

Therefore, this study aimed to screen and identify compounds with anti-PEDV activity and elucidate their antiviral mechanisms of action. Specifically, we performed high-throughput screening of 556 compounds and identified magnolol as a potent PEDV inhibitor both *in vitro* and *in vivo*. Further experiments were performed to elucidate the mechanisms underlying the antiviral activity of magnolol against PEDV. Overall, this study is anticipated to provide valuable insights into the antiviral effects and mechanisms of magnolol and offer potential targets for the prevention and treatment of viral infections in the swine industry.

## RESULTS

### Screening of anti-PEDV compounds

**Fig 1:**
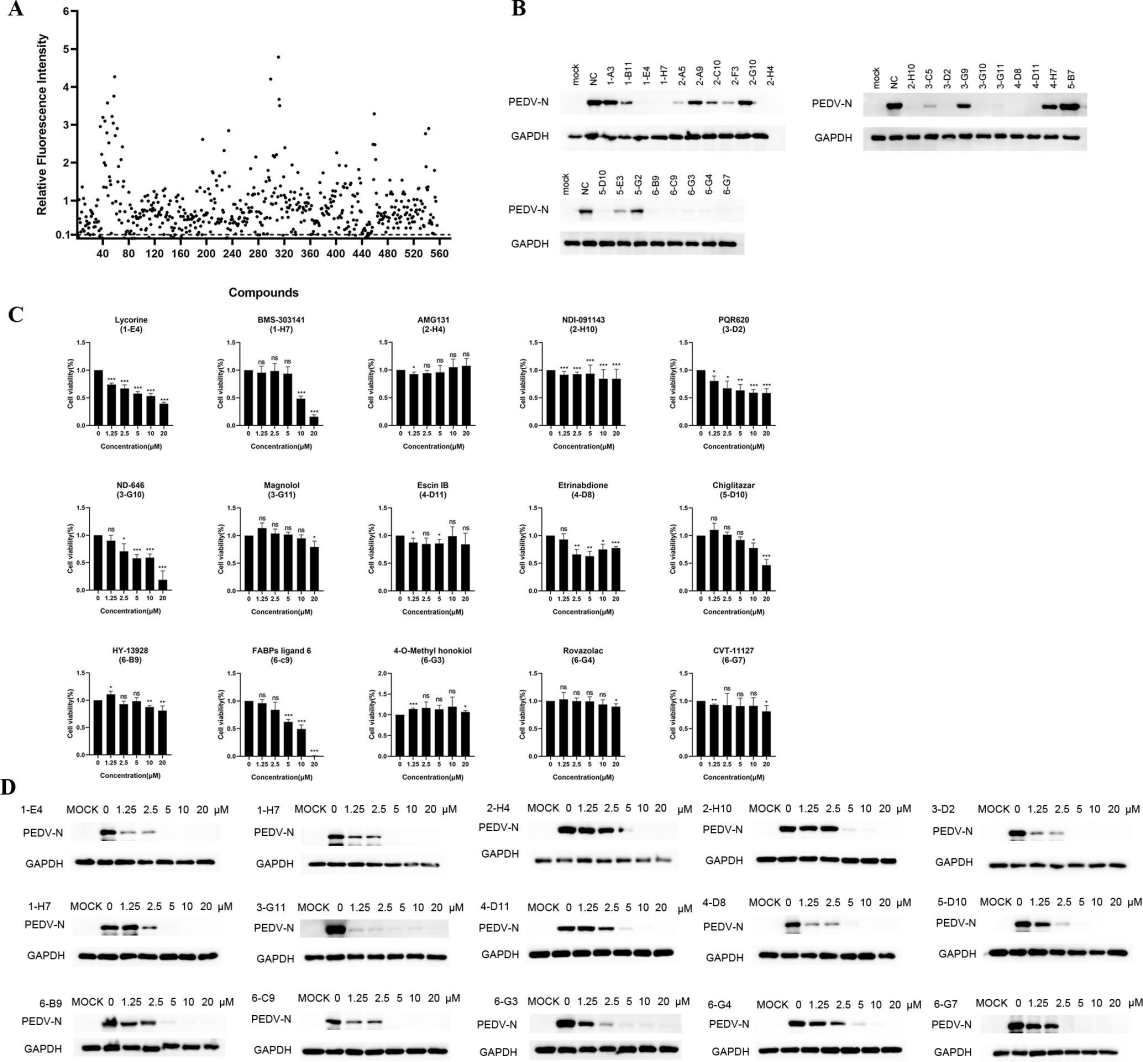
Screening of anti-PEDV compounds. (**A**) Primary screening of 556 compounds for inhibition of PEDV infection. Each dot represents the percentage of inhibition at a concentration of 5 μM. (**B**) Secondary screening of 25 candidate compounds by Western blot analysis. (**C**) Cytotoxicity of compounds at different concentrations determined by the CCK-8 assay. (**D**) Evaluation of the antiviral activity of low-toxicity compounds against PEDV by Western blotting.

Vero cells were used to screen and identify potential antiviral agents against PEDVs. Among the 556 compounds screened using in-cell Western (ICW) blotting analysis, 28 exhibited potent antiviral activity. Additionally, compounds showing no detectable PEDV N protein bands on Western blotting were selected for subsequent analysis. In total, 15 compounds were selected (Fig. 1B) and subjected to cytotoxicity assays. Additionally, the Cell Counting Kit-8 (CCK-8) assay indicated that eight compounds maintained cell viability above 80% (Fig. 1C). To identify the candidate drug with the strongest antiviral activity, we utilized Western blotting to detect the inhibitory capacity of these compounds against PEDV infection. The results showed that magnolol (3-G11) exhibited the most significant antiviral effect, reducing viral N protein expression by more than 90% at a concentration of 1.25 μΜ (Fig. 1D). In IPEC cells, magnolol showed antiviral activity comparable to or stronger than that of the other selected compounds, consistent with the results observed in Vero cells (Fig. S3).

### Dose-dependent inhibition of PEDV replication by magnolol

**Fig. 2:**
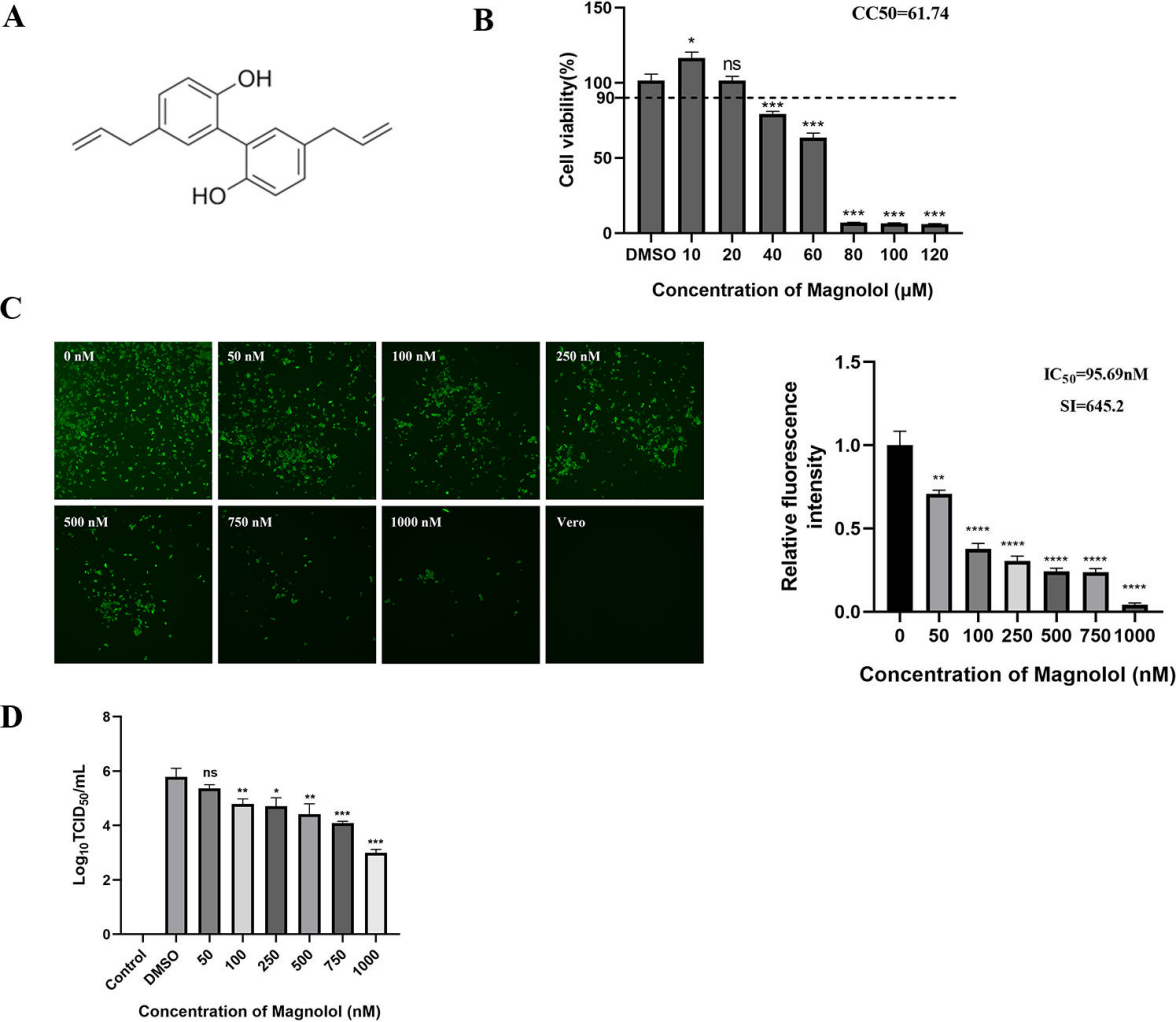
Magnolol inhibits PEDV infection in a dose-dependent manner. (**A**) Chemical structure of magnolol. (**B**) Vero cells were treated with different concentrations of magnolol (10, 20, 40, 60, 80, 100, and 120 μM). The relative viability of untreated cells (0 μM) was set as 100%. (**C**) Antiviral activity of magnolol at different concentrations was determined at 24 hpi using immunofluorescence assay, and fluorescence intensity was quantified with a multifunctional microplate reader. (**D**) The effects of various concentrations of magnolol on viral titers were measured using the TCID_50_ assay. Significance compared with the infection group is indicated as *P < 0.05, **P < 0.01, ***P < 0.001, and ****P < 0.0001.

Figure 2A shows the chemical structure of magnolol. To evaluate cytotoxicity, we expanded the concentration range of Vero cells. Magnolol exhibited almost no cytotoxicity at concentrations ≤20 µM, with a 50% cytotoxic concentration (CC_50_) value of 61.74 μM (Fig. 2B). Additionally, we assessed the antiviral activity of magnolol at 50, 100, 250, 500, 750, and 1,000 nM. Notably, magnolol treatment inhibited PEDV N protein expression in a dose-dependent manner, with a half-maximal inhibitory concentration of 95.69 nM in Vero cells, which was markedly lower than its CC_50_ (Fig. 2B and C). Additionally, the selectivity index was 645.2, indicating that magnolol is a potent anti-PEDV compound (Fig. 2C). Moreover, we examined the viral titers using the 50% tissue culture infectious dose (TCID_50_) assay and found that the viral titer decreased significantly under the same concentration gradient (Fig. 2D). Collectively, these findings demonstrate that magnolol significantly inhibits PEDV replication *in vitro*.

### Magnolol significantly inhibits the endocytic stage of PEDV infection

**Fig 3:**
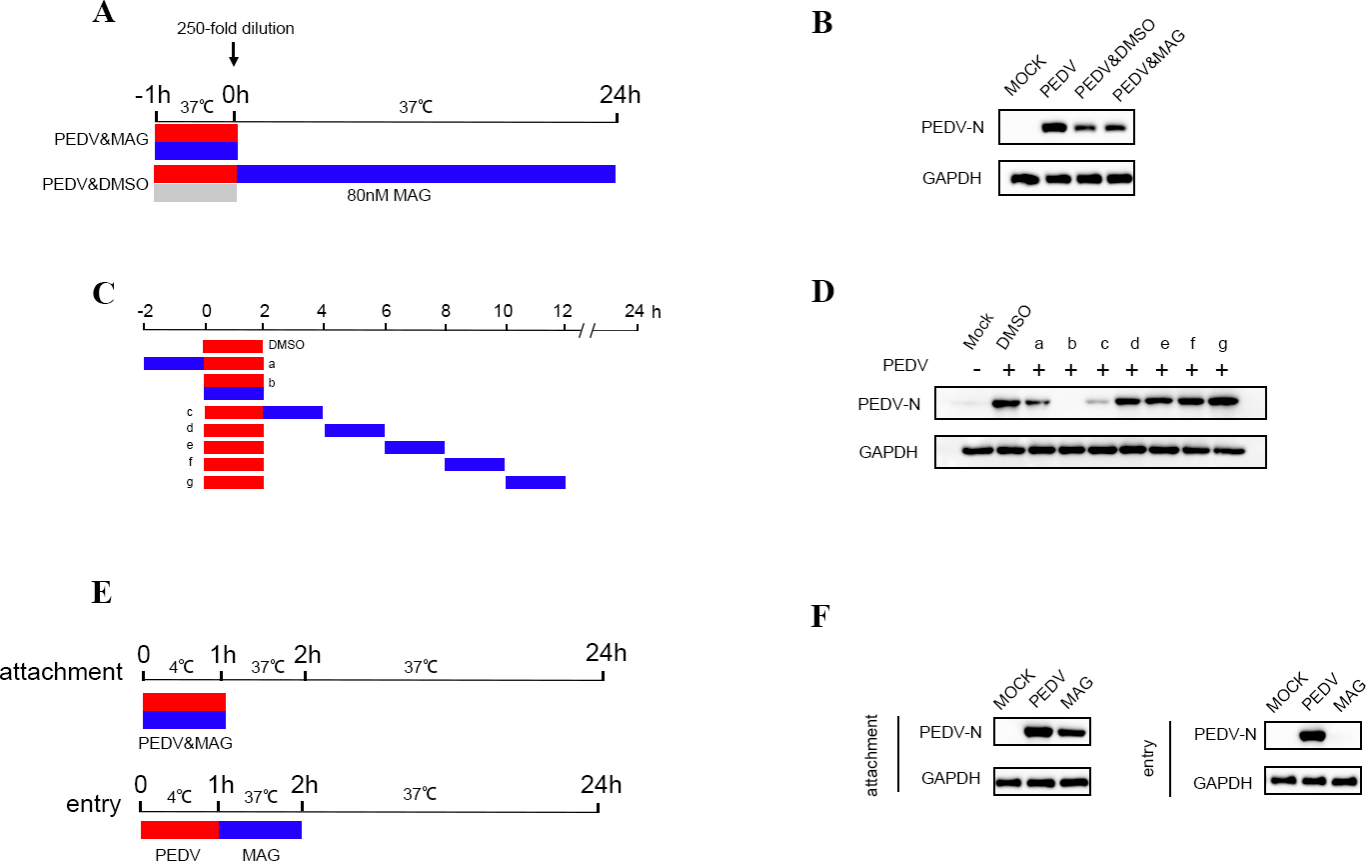
Magnolol inhibits PEDV infection at different stages of the viral life cycle. (**A**) Schematic diagram of the virus inactivation assay with magnolol. (**B**) Western blot analysis of the effects of magnolol–PEDV coincubation on PEDV infection in Vero cells. (**C**) Schematic representation of the time-of-addition assay. Red and blue bars indicate the time intervals of PEDV infection and magnolol treatment, respectively. (**D**) Samples were collected at 24 hpi for Western blot analysis. a, b, c, d, e, f and g correspond to the magnolol treatment intervals shown in panel C. (**E**) Schematic illustration of the viral adsorption and internalization assays. (**F**) Western blot analysis of the effects of magnolol on PEDV adsorption and internalization.

To determine whether magnolol exerted a direct virucidal effect on PEDV, we incubated magnolol (20 μM) with PEDV at a 1:1 ratio at 37°C for 1 h. In parallel, an equivalent volume of DMSO was incubated with PEDV under identical conditions as a solvent control. Thereafter, the mixture was diluted and used to infect Vero cells at a multiplicity of infection (MOI) of 0.1, followed by incubation at 37°C for 24 h. To exclude the potential influence of residual magnolol during infection, 80 nM magnolol was added to the solvent control during the infection period. Western blotting showed no significant difference in PEDV N protein expression between the magnolol-preincubated group and the DMSO-preincubated control (Fig. 3B), indicating that magnolol did not directly inactivate PEDV.

To identify the stage of the viral life cycle targeted by magnolol, a time-of-addition assay was performed using Vero cells (MOI = 0.1). As illustrated in Fig. 3C, magnolol was added at different time points relative to the viral infection. Magnolol treatment inhibited PEDV replication at multiple stages, with the strongest inhibitory effect observed at 0–2 h post-infection, suggesting that magnolol predominantly interfered with viral entry. Additionally, we examined whether magnolol affected viral adsorption or internalization (Fig. 3E) and found that magnolol exerted a pronounced inhibitory effect on the internalization stage of PEDV entry (Fig. 3F).

### Magnolol restricts PEDV to late endosomes

**Fig 4:**
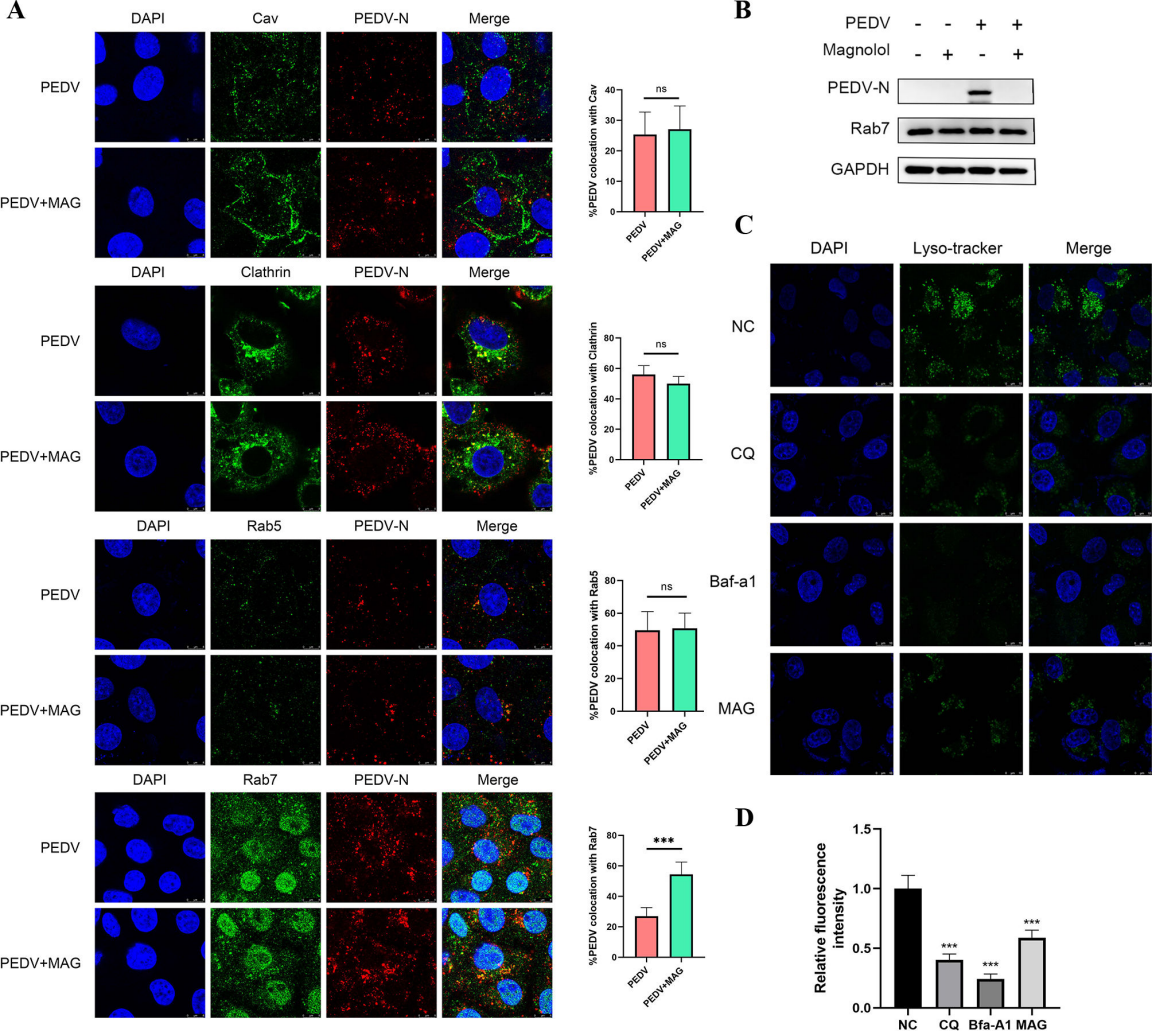
Effects of magnolol on the endocytic pathway. (**A**) Colocalization of PEDV N (red) with clathrin HC, caveolin-1, Rab5, and Rab7 (green) observed under a confocal microscope. (**B**) Western blot analysis of Rab7 protein expression in Vero cells after magnolol treatment. (**C and D**) IPEC cells were seeded in confocal dishes and exposed to PEDV (MOI = 0.1) at 4°C for 1 h. After washing three times, cells were incubated with DMSO, CQ, Baf-A1, or magnolol dilution at 37°C for 1 h. Cells were then stained with 50 nM LysoTracker Green for 30 min. Green fluorescence indicates acidic organelles. Significance compared with the infection group is indicated as ns (*P* > 0.05) and ***P < 0.001.

PEDV enters cells via clathrin- and caveolae-mediated endocytosis. After internalization, the virus is transported through early and late endosomes to lysosomes, where membrane fusion releases the viral genome into the cytoplasm. To investigate the effect of magnolol on PEDV entry, the localization of PEDV and virus entry-related proteins was examined at various time points after magnolol treatment. As shown in Fig. 4A, the colocalization of PEDV particles with caveolin, clathrin, and Rab5 showed no significant differences between the virus-infected and magnolol-treated groups. In contrast, colocalization with Rab7 markedly increased following magnolol treatment, indicating that PEDV was trapped in late endosomes. However, Western blotting analysis results confirmed that magnolol did not significantly alter Rab7 protein expression (Fig. 4B).

Previous studies have shown that most PEDV particles are transported to Rab7-positive endosomes for viral fusion (24). Viral genome release depends on endosomal acidification during this process. Therefore, we hypothesized that magnolol restricts PEDV to late endosomes by inhibiting endosomal acidification, thereby preventing membrane fusion and cytoplasmic entry of the virus. Chloroquine (CQ), an inhibitor of endosomal acidification, and bafilomycin A1 (Baf-A1), a V-ATPase inhibitor that blocks cargo transport from early to late endosomes and destabilizes the low pH environment of lysosomes, were used as controls. IPEC cells pretreated with chloroquine, Baf-A1, or magnolol were infected with PEDV, and endosomal acidification was evaluated using LysoTracker Green. Staining with 50 nM LysoTracker Green revealed that treatment with 20 μM magnolol significantly inhibited endosomal acidification (Fig. 4C and D).

### Magnolol inhibits CTSL expression in a dose-dependent manner

**Fig 5:**
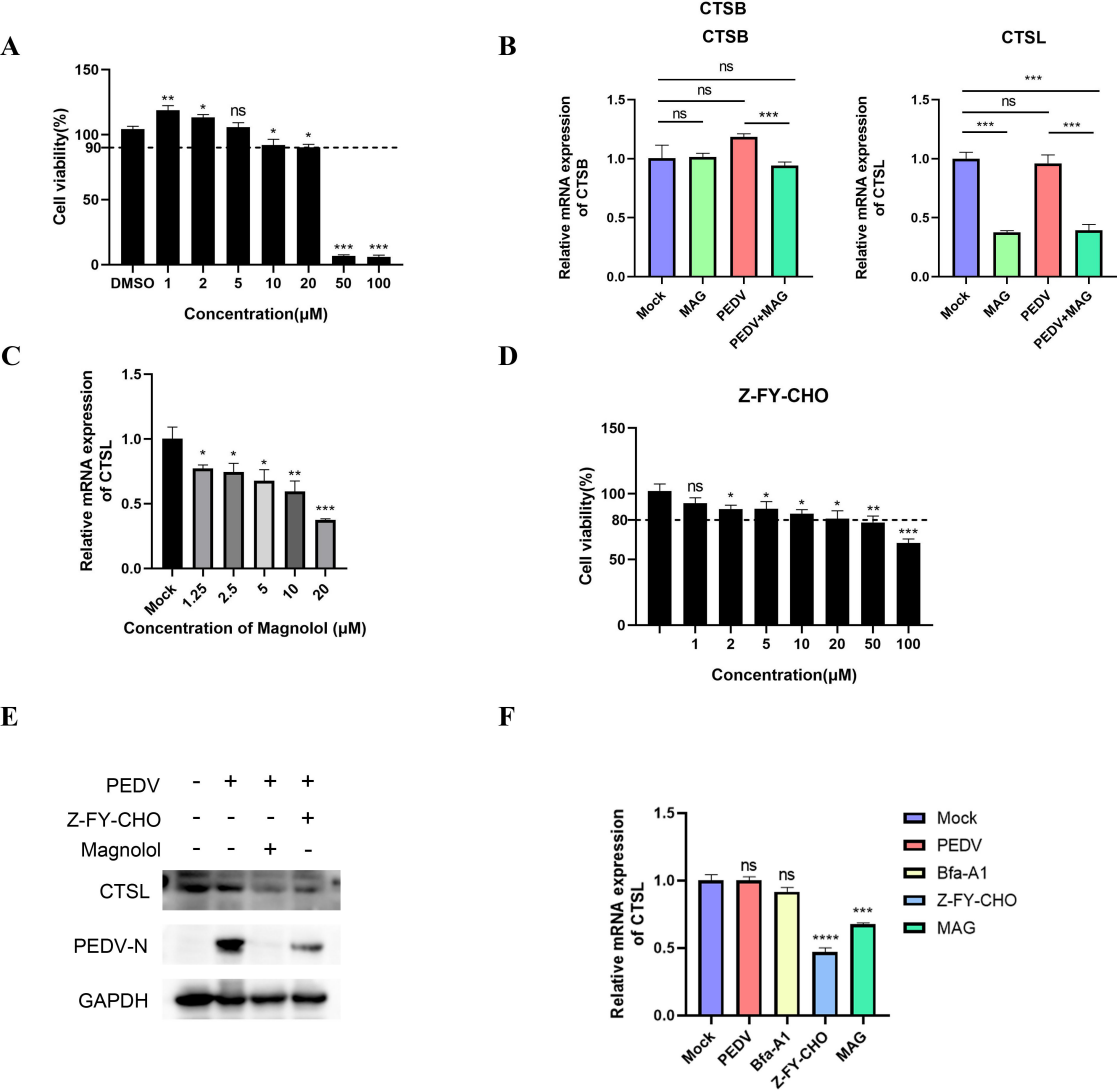
Magnolol inhibits CTSL expression. (**A**) Cytotoxicity of magnolol on IPEC cells was evaluated using the CCK-8 assay. (**B**) Total RNA was collected and analyzed by RT-qPCR to determine the effects of magnolol on CTSB and CTSL expression. (**C**) RT-qPCR analysis of CTSL mRNA levels in IPEC cells treated with different concentrations of magnolol. (**D**) Cytotoxicity of Z-FY-CHO on IPEC cells was assessed using the CCK-8 assay. (**E**) IPEC cells were exposed to PEDV at 4°C for 1 h, then incubated with Z-FY-CHO or magnolol dilutions at 37°C for 1 h. After 24 h, cells were harvested for Western blot analysis of PEDV N and CTSL protein levels. (**F**) RT-qPCR analysis of CTSL mRNA levels in cells treated with Baf-A1, Z-FY-CHO, or magnolol. Significance compared with the infection group is indicated as ns (*P* > 0.05), *P < 0.05, **P < 0.01, ***P < 0.001, and ****P < 0.0001.

Previous studies have revealed that PEDV entry requires the activation of lysosomal-cysteine proteases. First, we evaluated the cytotoxicity of magnolol in IPEC and determined that 20 μM was suitable for further experiments. To investigate the effect of magnolol on cathepsins, RT-qPCR was performed to determine the expression levels of CTSL and CTSB after magnolol treatment. RT-qPCR analysis revealed that CTSB mRNA levels remained unchanged in the magnolol-only group but were significantly downregulated in the treatment group compared with the virus-infected group. In contrast, CTSL mRNA expression was markedly downregulated in both the magnolol-only and treatment groups (Fig. 5B).

Additionally, we examined the dose-response relationship and found that magnolol suppressed CTSL mRNA expression in a dose-dependent manner (Fig. 5C). To confirm this at the protein level, we examined CTSL protein expression in cells treated with magnolol and the CTSL inhibitor Z-FY-CHO (20 μM) using Western blotting. Notably, both treatments significantly reduced PEDV replication and downregulated CTSL expression (Fig. 5D).

Given that magnolol inhibited endosomal acidification (Fig. 4C and D) and that the acidic environment of endosomes and lysosomes is required for CTSL activation, we investigated whether the reduction in CTSL expression was caused by changes in endosomal pH. Specifically, CTSL expression was examined under different treatment conditions, with Baf-A1-treated cells serving as a positive control. Notably, magnolol-mediated suppression of CTSL was not dependent on changes in endosomal acidification, as Baf-A1 treatment did not alter CTSL expression (Fig. 5F).

### Magnolol ameliorates tissue damage and improves survival in infected piglets

**Fig 6:**
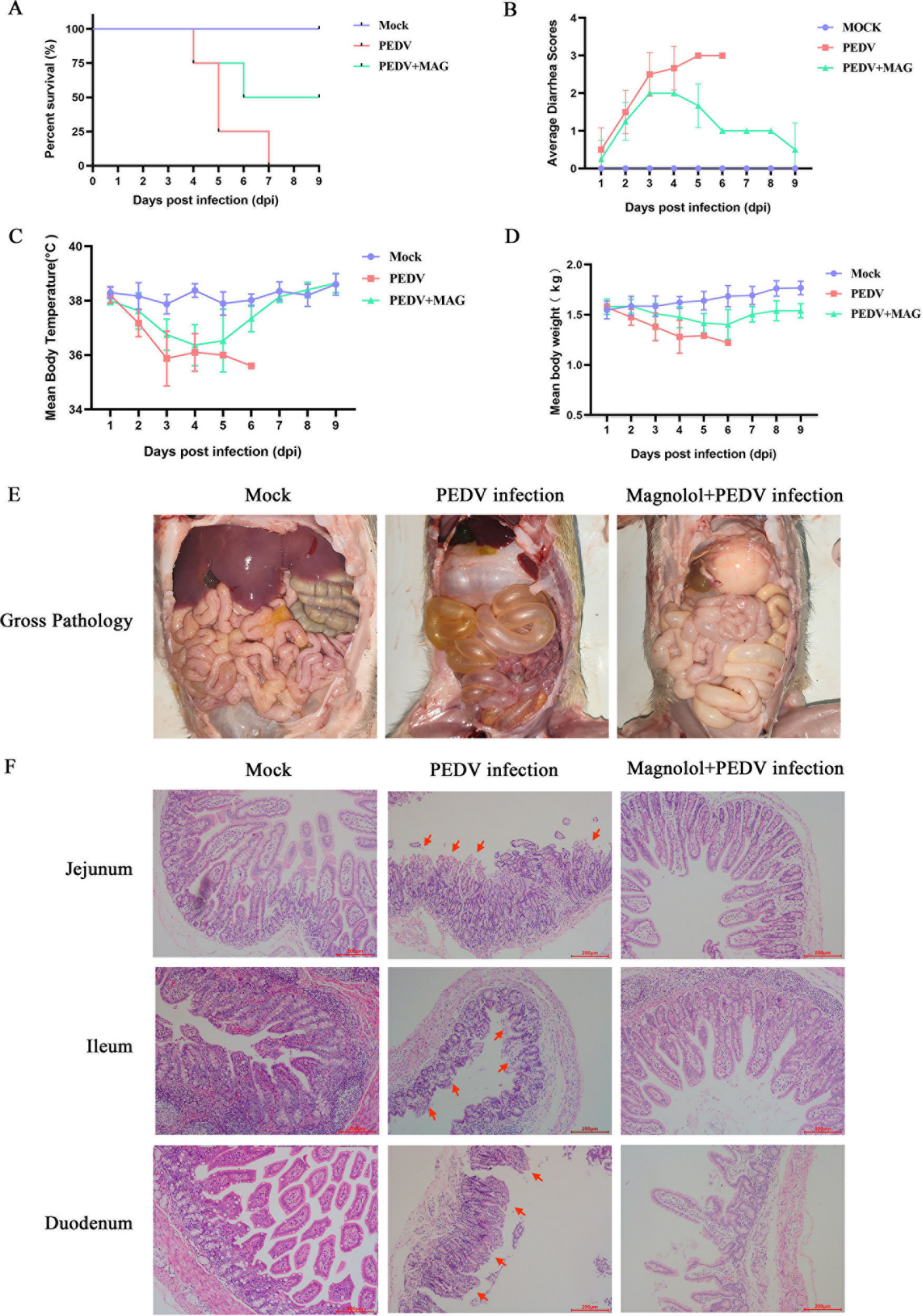
Clinical symptoms and histopathological analysis of piglets. (**A**) Survival rate of piglets. (**B**) Diarrhea scores of piglets, where 0 = normal, 1 = soft feces, 2 = loose feces, and 3 = watery diarrhea. (**C**) Changes in rectal temperature of piglets. (**D**) Changes in body weight of piglets. (**E**) Clinical manifestations and gross intestinal lesions in suckling piglets. (**F**) Histopathological sections of jejunum, ileum, and duodenum tissues.

*In vivo* experiments were performed to investigate the antiviral efficacy of magnolol in 3-day-old piglets infected with PEDV. In the PEDV-infected group, diarrhea appeared at 1 day post-infection (dpi) and peaked at 5 dpi. Clinical signs include hypothermia, emaciation, and a high mortality rate. In contrast, diarrhea began at 1 dpi and peaked at 3–4 dpi (presented as loose stools) in the magnolol-treated group. Additionally, the decrease in body temperature was slower than that observed in the PEDV-infected group, reaching its lowest level at 4 dpi. Moreover, weight loss was less pronounced in the magnolol-treated group, and the piglets started regaining weight at 7 dpi. Expectedly, mock-infected piglets remained healthy throughout the study period (Fig. 6B through D).

Piglet deaths in both the PEDV-infected and magnolol-treated groups began at 4 dpi. At 7 dpi, the survival rates were 0%, 50%, and 100% in the PEDV, magnolol, and mock groups, respectively. At 10 dpi, all piglets were euthanized for necropsy and histopathological examinations (Fig. 6A). Gross pathology revealed normal intestinal morphology with no visible lesions in the mock group. In contrast, piglets in the PEDV-infected group displayed severe intestinal lesions and morphological changes, including thinning of the intestinal wall, distension of the lumen with gas, and yellow fluid accumulation. However, piglets in the magnolol-treated group exhibited markedly attenuated lesions, with less severe intestinal distension and reduced luminal gas accumulation (Fig. 6E).

Histopathological analyses of the jejunum, ileum, and duodenum confirmed these findings. PEDV-infected piglets exhibited severe pathological changes, including nearly complete villous atrophy of the jejunum and duodenum, ileal hemorrhage and vacuolation, and shortened villi. In comparison, piglets in the magnolol-treated group displayed less severe intestinal pathology. Collectively, these results indicate that magnolol treatment effectively alleviates PEDV-induced clinical symptoms and intestinal injury, thereby improving the survival rate of infected piglets (Fig. 6F).

### Magnolol significantly reduces viral load in rectal swabs and intestinal tissues

**Fig 7:**
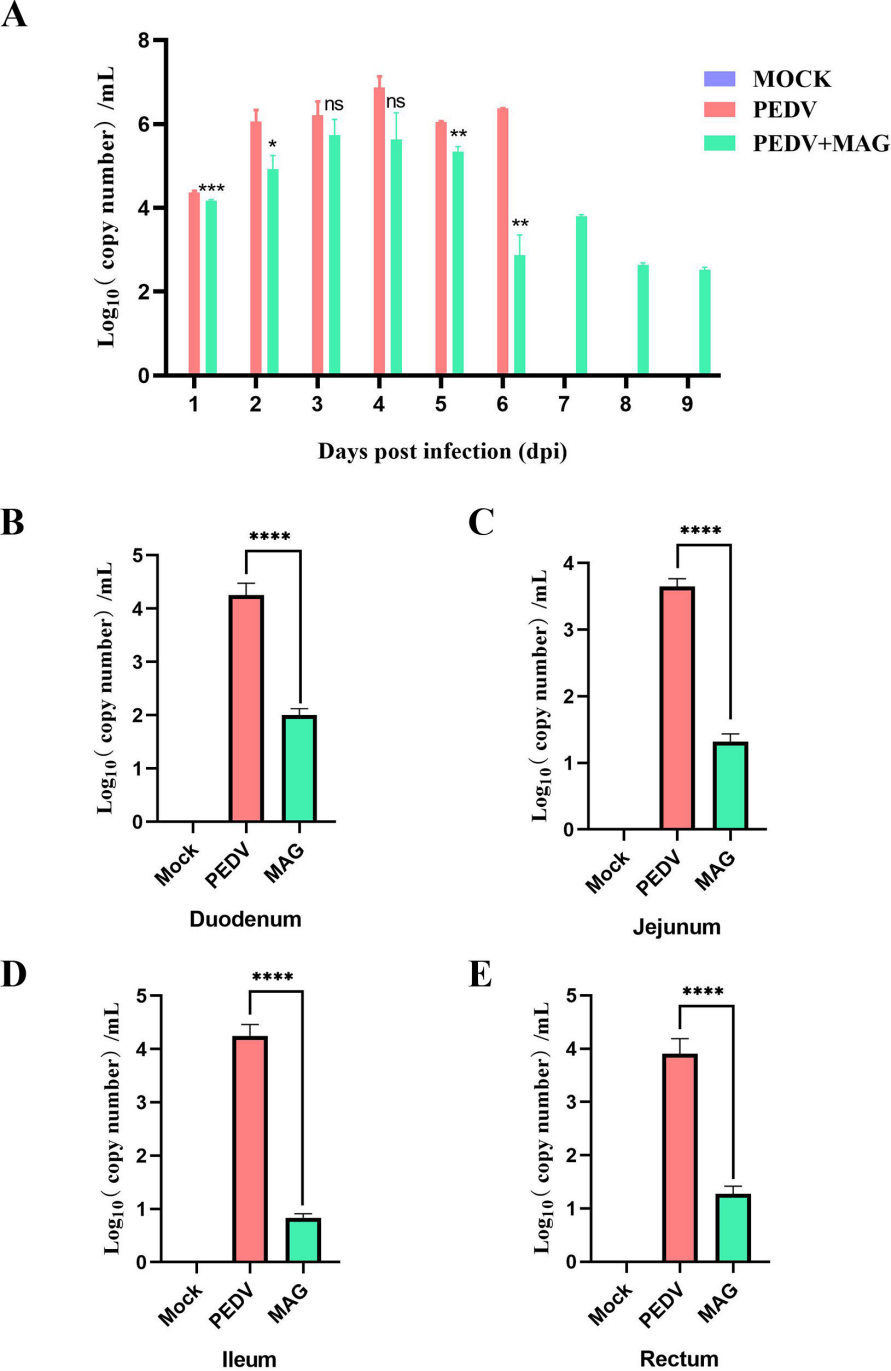
Viral loads in intestinal tissues and anal swabs. (**A**) Viral loads in anal swabs of piglets from the mock, PEDV, and magnolol-treated groups measured by RT-qPCR. (**B–E**) Viral loads in duodenum, jejunum, ileum, and rectum of piglets from the mock, PEDV, and magnolol-treated groups measured by RT-qPCR. Significance compared with the infection group is indicated as ns (*P* > 0.05), *P < 0.05, **P < 0.01, ***P < 0.001, and ****P < 0.0001.

RT-qPCR was performed to assess the viral load in rectal swabs and intestinal tissues. Compared with the PEDV-challenged group, the magnolol-treated group showed a significant reduction in viral load at 1 and 2 dpi. At 3–4 dpi, the peak period of diarrhea (Fig. 6B), no significant difference was observed in viral load between the two groups. However, viral load was significantly lower in the treatment group than in the challenge group in the following days (Fig. 7A). Additionally, we assessed viral loads in different intestinal tissues, including the duodenum, jejunum, ileum, and rectum. Notably, the PEDV-challenged group exhibited substantially higher viral loads in all examined tissues than the magnolol-treated group (Fig. 7B through E). Overall, these findings indicate that magnolol exerts significant antiviral effects against PEDV *in vivo*.

### Magnolol reduces CTSL expression in the intestine and alters its localization in intestinal epithelial cells

**Fig 8:**
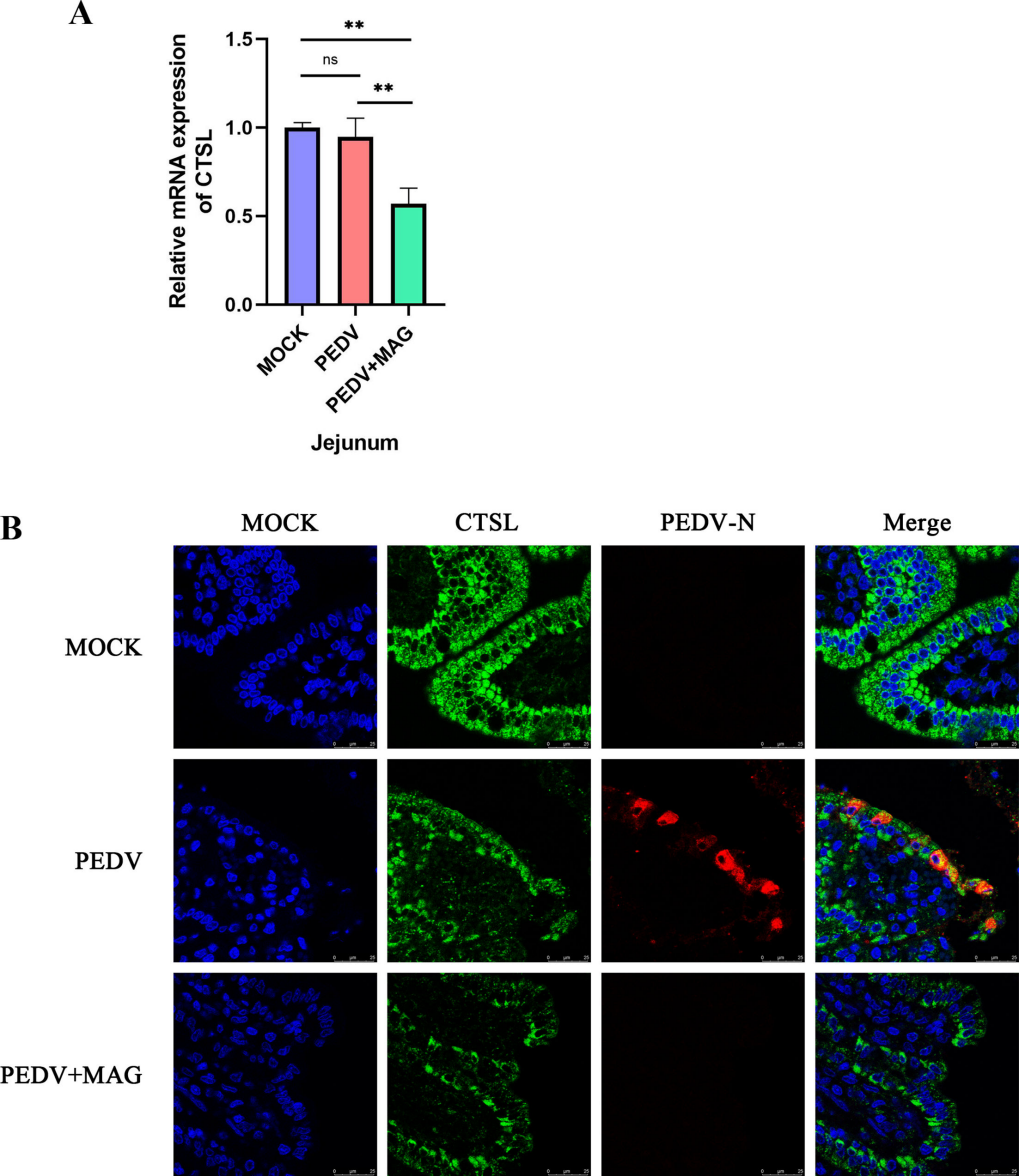
Effect of magnolol treatment on CTSL in the intestines of PEDV-infected piglets. (**A**) CTSL mRNA levels in the jejunum of piglets from the mock, PEDV, and magnolol-treated groups measured by RT-qPCR. (**B**) Immunofluorescence double staining of jejunum tissue from piglets. Confocal microscopy was used to observe the distribution of PEDV N and CTSL in intestinal tissues. White and red arrows indicate the distal and proximal regions of the jejunal epithelium, respectively. Significance compared with the infection group is indicated as ns (*P* > 0.05), **P < 0.01.

Our previous results demonstrated that magnolol downregulates CTSL expression in PEDV-infected IPEC. To further investigate whether magnolol affects CTSL expression in intestinal tissues, RT-qPCR was performed to assess CTSL mRNA expression in piglet intestinal tissues. CTSL mRNA levels were significantly lower in the magnolol-treated group than in the PEDV-challenged group (Fig. 8A). Immunofluorescence analysis revealed that magnolol treatment markedly decreased the fluorescence intensity of PEDV N protein. In Fig. 8B, the distal and proximal regions of the jejunal epithelium are indicated by white and red arrows, respectively. CTSL expression was significantly lower in the distal region than in the proximal region after treatment with magnolol. In contrast, CTSL expression did not differ between the distal and proximal epithelial cell regions in the PEDV-challenged group (Fig. 8B). Overall, these findings suggest that magnolol inhibits CTSL expression in intestinal epithelial cells *in vivo*, thereby preventing PEDV infection.

### Magnolol inhibits PDCoV and PEAV replication

**Fig 9:**
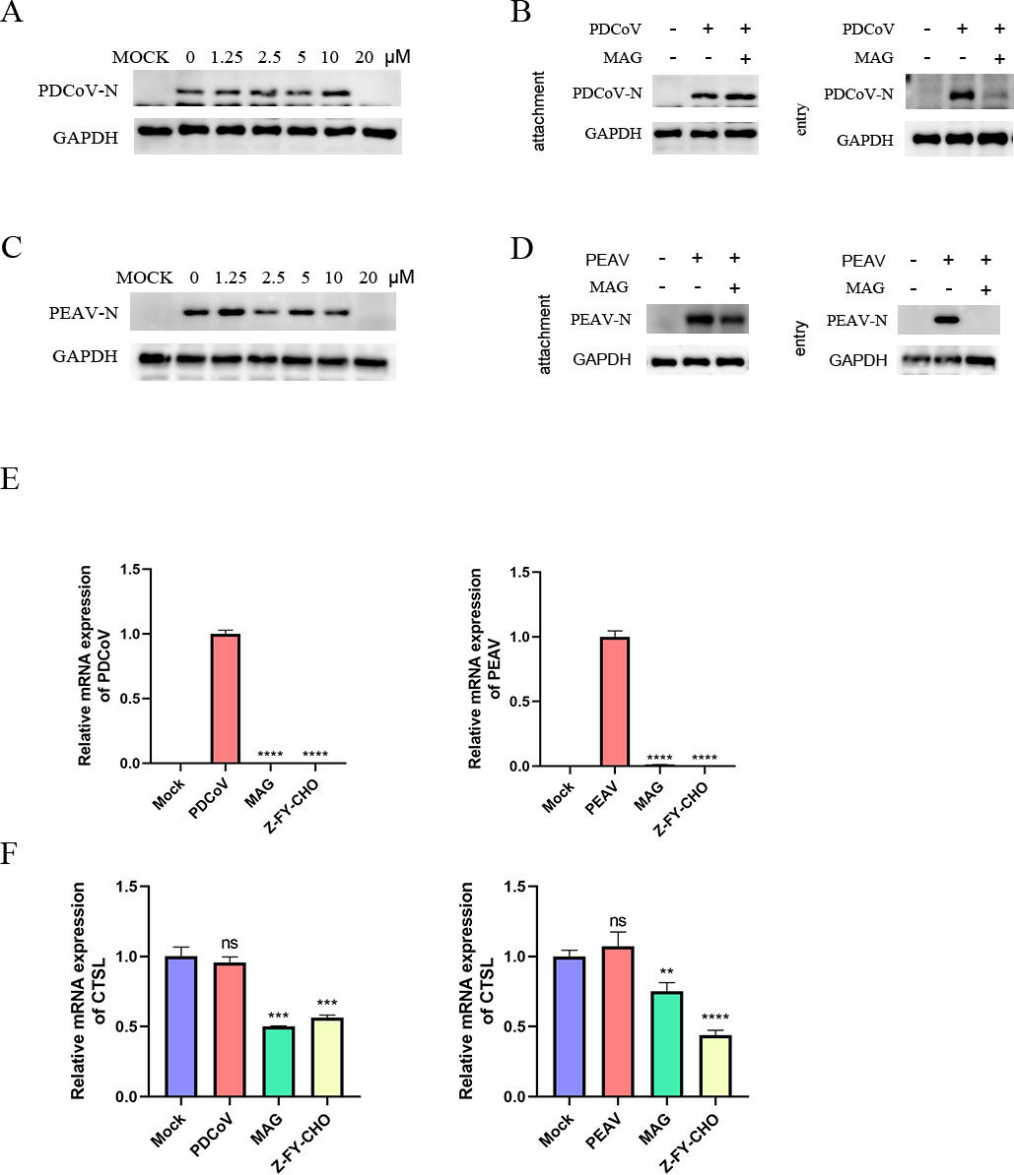
Magnolol inhibits PDCoV and PEAV infection in LLC-PK cells. (**A**) Western blot analysis of the effect of different concentrations of magnolol on PDCoV infection. (**C**) Western blot analysis of the effect of different concentrations of magnolol on PEAV infection. (**B and D**) Effects of magnolol on adsorption and internalization of PDCoV and PEAV. (**E**) RT-qPCR analysis of PEAV and PDCoV N gene mRNA levels. (**F**) RT-qPCR analysis of CTSL mRNA levels after treatment with magnolol or Z-FY-CHO. Significance compared with the infection group is indicated as ns (*P* > 0.05), **P < 0.01, ***P < 0.001, and ****P < 0.0001.

To investigate the broad-spectrum antiviral activity of magnolol against other porcine coronaviruses, we examined its effects on the replication of porcine deltacoronavirus (PDCoV) and porcine enteric alphacoronavirus (PEAV). Western blot analysis of infected LLC-PK cells revealed that treatment with 20 μM magnolol effectively suppressed the replication of PDCoV and PEAV (Fig. 9A and C). Time-of-addition assays revealed that magnolol significantly inhibited the endocytic stages of PDCoV and PEAV infections (Fig. 9B and D). Additionally, we investigated whether magnolol regulates CTSL expression in cells infected with PDCoV or PEAV. Treatment with CTSL inhibitor and magnolol markedly reduced viral replication and downregulated CTSL expression (Fig. 9E and F). Moreover, magnolol treatment significantly increased the colocalization of the viral N protein with the late endosomal marker Rab7 in PDCoV- and PEAV-infected IPEC cells, suggesting that viral particles were trapped in late endosomes and failed to undergo endosomal escape (Fig. S5). Collectively, these findings suggest that magnolol exerts broad-spectrum antiviral activity against porcine coronaviruses by targeting CTSL-dependent viral entry.

## DISCUSSION

Porcine epidemic diarrhea (PED), caused by PEDV, is a highly contagious enteric disease in pigs. PEDV infection results in severe diarrhea in piglets, particularly in neonates, with mortality rates as high as 80%–100%. As newborn piglets lack sufficient time to receive vaccinations and develop protective immunity, they primarily rely on maternal antibodies in colostrum and milk to defend against PEDV (25). Moreover, the high genetic variability of the PEDV S gene has led to the emergence of multiple genotypes (26) and low levels of vaccine-induced secretory IgA in milk (25, 27), which are likely responsible for the limited protective efficacy of current commercial PEDV vaccines.

In this study, we screened 556 compounds and identified several with >90% inhibitory activity against PEDV. Among them, magnolol exhibited the most potent antiviral activity. 4-O-Methylmagnolol and magnolol (6-G3), lignan compounds isolated from *Magnolia officinalis*, exhibited notable inhibitory activity (Fig. 1D). An apparent discrepancy was observed between the pronounced reduction in N protein expression (Fig. 1D) and the relatively modest decrease in viral titers (Fig. 2D). This difference can be explained by the stage at which magnolol exerts its antiviral activity. Subsequent mechanistic experiments demonstrated that magnolol primarily interferes with the viral entry process (Fig. 3F), thereby limiting the number of cells initially infected. Nevertheless, a small proportion of viruses that successfully enter host cells may continue to replicate and accumulate over time, resulting in measurable viral titers at later time points. *In vivo* experiments were performed to validate the anti-PEDV efficacy of magnolol in 3-day-old piglets. Oral administration of magnolol significantly improved the survival rate of infected piglets. Regarding the *in vivo* efficacy, although magnolol treatment increased the survival rate of PEDV-infected piglets by approximately 50%, this level of protection is comparable to, or even higher than, that reported for several previously described anti-PEDV candidate compounds. Notably, many reported antiviral candidates did not include survival outcomes (28, 29) or employed infection models in which mortality occurred at later stages of infection (9–10 days post-infection), indicating relatively low viral virulence (30). In contrast, the PEDV strain used in this study caused substantial mortality as early as 4 days post-infection, representing a more stringent infection model. In addition, magnolol exhibited a higher selectivity index and stronger antiviral activity *in vitro* than many previously reported anti-PEDV compounds (31–33). The moderate protective efficacy observed *in vivo* may be partially attributable to the limited aqueous solubility of magnolol, a known pharmacokinetic limitation that may restrict its bioavailability. Importantly, this limitation could potentially be addressed through further formulation optimization. Collectively, these findings support magnolol as a promising antiviral lead compound against PEDV rather than a fully optimized therapeutic agent. Moreover, magnolol treatment markedly reduced viral loads in rectal swabs and intestinal tissues and alleviated PEDV-induced pathological changes, including intestinal wall thinning and transparency, intestinal distension with gas, and villous damage. In this study, magnolol was administered orally at a dose of 100 mg/kg. Although a magnolol-only control group was not included, this dose is substantially lower than those previously reported to be well tolerated in multiple animal species. Specifically, magnolol has been administered at 400 mg/kg in pigs, 100–400 mg/kg in chickens, and at repeated daily oral doses of 625–2,500 mg/kg in mice, without reports of mortality, overt clinical toxicity, or major histopathological alterations in vital organs (34–36). Collectively, these studies indicate a relatively wide safety margin for magnolol. Nevertheless, we acknowledge that a dedicated safety evaluation of magnolol in neonatal piglets, including systematic clinical observations and histopathological analyses, would provide stronger support for its potential use. Such studies will be an important focus of future work, particularly in the context of formulation optimization to improve bioavailability and reduce the required effective dose.

The PEDV life cycle includes attachment, entry, replication, and release phases. Contrary to the findings of Wang et al. (37), magnolol exhibited antiviral activity at both the entry and replication stages of PEDV, with a more pronounced inhibitory effect during the entry stage. Notably, we speculate that this discrepancy may be attributable to differences in stock solution preparation. During our experiments, we found that a 100 mM stock solution tended to precipitate upon serial dilution, resulting in incomplete solubilization and therefore an effectively lower working concentration than intended. Magnolol exerts diverse biological activities, including anti-inflammatory, antioxidant, and metabolic regulatory effects, and modulates signaling pathways, such as NF-κB/MAPK and PI3K/Akt/mTOR, to inhibit ROS generation and regulate glucose and lipid metabolism (38). Previous studies have demonstrated that PEDV induces lipid droplet accumulation and activates NF-κB signaling (39), whereas inhibition of the PI3K/Akt signaling pathway suppresses PEDV replication (40). Mu et al. reported that magnolol reduces the formation of lipid droplets (41). Overall, these findings may explain the clear inhibitory effect of magnolol at the replication stage. Our study revealed that magnolol suppressed PEDV-induced upregulation of TNFα, IL-6, and IL-8 in Vero cells and reduced Akt phosphorylation after PEDV infection (Fig. S1). Given that magnolol had the most significant effect during the internalization step, we prioritized characterization at this stage and did not pursue further mechanistic studies on replication.

After clathrin-mediated endocytosis, viruses reach early endosomes within 2 min, are subsequently trafficked to multivesicular and late endosomes, and ultimately enter lysosomes within 30–60 min (42). Liu et al. reported that PEDV enters host cells via clathrin- and caveolin-mediated endocytic pathways and is transported to endosomes and lysosomes (9). In the present study, we monitored PEDV colocalization with caveolin, clathrin, Rab5, and Rab7 using confocal microscopy at different time points after magnolol treatment. As shown in Fig. 4A, PEDV and Rab7 colocalization increased; however, Western blotting indicated that magnolol did not alter Rab7 protein levels. Given that PEDV entry depends on the acidic environment of endosomes (43, 44), we hypothesized that magnolol interferes with endosomal acidification, thereby blocking PEDV trafficking to lysosomes or preventing membrane fusion with the cytoplasm of the host cell. Consistent with our hypothesis, magnolol significantly inhibited endosomal acidification in a manner comparable to that of chloroquine and Baf-A1 (Fig. 4B and C).

Membrane fusion requires a conformational rearrangement of viral glycoproteins. Unlike influenza and dengue viruses, a low pH alone is insufficient to trigger PEDV S-mediated fusion (43); additional proteolytic activation is required. Liu et al. identified the cathepsins CTSL and CTSB as essential for the proteolytic activation of PEDV S protein during viral entry (9). Our study showed that magnolol treatment significantly and dose-dependently downregulated CTSL expression, whereas CTSB levels remained unchanged (Fig. 5B, C and F). Given that cathepsin activation requires an acidic environment, we used Baf-A1 as a control to determine whether CTSL downregulation was due to impaired acidification. *In vitro*, magnolol-induced CTSL downregulation was independent of endosomal acidification. Similarly, magnolol treatment markedly downregulated CTSL expression in the distal region of villous epithelial cells facing the intestinal lumen compared with that in the proximal region, near the lamina propria. In contrast, PEDV infection alone did not alter the distribution of CTSL between the distal and proximal epithelial regions. Overall, these findings suggest that magnolol suppresses PEDV infection by selectively reducing CTSL expression in villous epithelial cells. The apical side of intestinal epithelial cells, which faces the intestinal lumen, is the primary site of PEDV invasion in host cells. Based on these results, we speculate that magnolol reduces CTSL expression in the apical region, thereby limiting the proteolytic activation required by the virus during its initial contact with host cells, which impedes viral entry and replication.

To further explore whether magnolol directly interacts with CTSL rather than merely reducing its expression, we performed molecular docking analysis. Molecular docking of Z-FY-CHO and magnolol with CTSL revealed that both compounds occupied similar binding regions within the active site of the enzyme (Fig. S2). Importantly, the biphenolic scaffold of magnolol was positioned near the catalytic core formed by Cys25 and His163. Further analysis indicated that the aromatic ring of magnolol predominantly occupied the S2 subsite and partially extended into the S1 subsite. In contrast, its allyl side chain projects into the S3 region, suggesting that it may impede PEDV from accessing the catalytic center. At the molecular level, the phenolic hydroxyl groups of magnolol form hydrogen bonds with Cys25, Met161, Asp162, and Gly164. Among these, Cys25 is the catalytic core residue of CTSL, whereas Met161, Asp162, and Gly164 are located within or adjacent to the S2 subsite. Z-FY-CHO also formed hydrogen bonds with Cys25 and Asp162. Collectively, these findings suggest that magnolol competes with PEDV for the binding site, thereby interfering with the proteolytic process required for viral membrane fusion.

Considering that cathepsins have been implicated in the entry of multiple viruses, we evaluated whether magnolol could inhibit other porcine coronaviruses. Magnolol treatment significantly inhibited PEAV and PDCoV entry and downregulated CTSL expression. Consistent with the findings of Zhang et al., who demonstrated that both CTSL and CTSB activate PDCoV entry and that CTSL knockdown markedly inhibits PDCoV infection (45), treatment with magnolol and the CTSL inhibitor Z-FY-CHO suppressed CTSL expression and PDCoV-N protein levels in the present study. Notably, we provide the first evidence that CTSL inhibition strongly reduces PEAV infection. However, the precise mechanism by which magnolol regulates CTSL expression remains to be elucidated.

Magnolol, a primary bioactive compound extracted from the bark of *Magnolia officinalis*, benefits from a well-established purification process and exhibits high safety, with an oral median lethal dose (LD_50_) of >50 g/kg (11). Magnolol exerts inhibitory effects against multiple viruses, protects the intestinal barrier, and possesses significant anti-inflammatory and antioxidant properties, thereby mitigating viral infection-induced inflammation (38, 46). Our study showed that magnolol treatment downregulated CTSL expression *in vitro* and *in vivo*, inhibited PEDV infection, and suppressed PDCoV and PEAV infection *in vitro*. Collectively, these findings suggest that magnolol has considerable potential as an antiviral agent against porcine coronaviruses. Nevertheless, its poor water solubility and limited oral bioavailability represent important pharmacokinetic constraints that may restrict systemic exposure. Notably, recent studies have reported strategies to improve the oral bioavailability of magnolol, including formulation-based approaches and intravenous nanoemulsion delivery systems, supporting its translational potential (47, 48). Although further optimization is required prior to large-scale application, the combination of strong *in vitro* antiviral activity, acceptable *in vivo* efficacy, potential broad-spectrum activity against porcine enteric coronaviruses, and relatively low production cost indicates that magnolol represents a feasible and potentially cost-effective antiviral candidate for porcine use.

### Conclusion

In this study, we identified magnolol as a potent inhibitor of PEDV. Our results demonstrate that magnolol blocks PEDV entry by suppressing CTSL expression. *In vivo* experiments confirmed the significant antiviral activity of magnolol, which altered the intestinal distribution of CTSL in piglets and prevented PEDV infection of intestinal villous epithelial cells. Moreover, magnolol exhibited inhibitory effects against PEAV and PDCoV and downregulated CTSL expression during viral infections. Collectively, these findings suggest that magnolol is a promising and safe antiviral candidate for treating PED and diseases caused by other porcine coronaviruses.

## MATERIALS AND METHODS

### Cells, virus infection, and piglets

Vero-E6, LLC-PK, and IPEC cells were stored in the Department of Infectious Diseases, College of Veterinary Medicine, South China Agricultural University. Cells were cultured in Dulbecco’s modified Eagle medium (DMEM; Invitrogen, New York, USA) supplemented with 10% fetal bovine serum (Gibco, Invitrogen) at 37°C under a 5% CO_2_ atmosphere. At 100% confluency, the cells were washed three times with PBS and infected with viruses in DMEM containing 10 μg/mL trypsin (PEDV and PEAV) or 5 μg/mL trypsin (PDCoV) (Gibco, Billings, MO, USA). Twelve piglets (3 days old) that were negative for African swine fever virus, pseudorabies virus, porcine reproductive and respiratory syndrome virus, and PEDV antigens and antibodies were used for *in vivo* experiments.

### Antibodies, inhibitors, and reagents

GAPDH (mouse mAb, clone 1E6D9), RAB7 (rabbit polyclonal), and CTSL (rabbit polyclonal) antibodies were purchased from Proteintech. Anticlathrin heavy-chain (rabbit polyclonal), caveolin-1 (rabbit polyclonal), and RAB5 (rabbit polyclonal) antibodies were purchased from Abcam. Mouse monoclonal anti-PEDV/PEAV/PDCoV N antibodies were generated in our laboratory. Secondary antibodies conjugated to Alexa Fluor 488 or Alexa Fluor 594 were purchased from Proteintech. Goat antirabbit/mouse IgG H&L (HRP) were supplied by Abcam; bafilomycin-A1 (S1413) was purchased from Selleck Chemicals; chloroquine phosphate (PHR1258) was purchased from Sigma-Aldrich; magnolol was purchased from Macklin; and CCK-8 was purchased from Beyotime Biotechnology (China). The lipid Metabolism Compound Library and Z-FY-CHO were purchased from MedChemExpress.

### ICW

Vero cells were grown in 96-well plates and infected with PEDV at 37°C for 1 h. After washing, the cells were incubated with different compound dilutions at 37°C for 24 h in a 5% CO_2_ atmosphere. Thereafter, the cells were washed, fixed with 4% paraformaldehyde, permeabilized with Triton X-100, and stained with specific antibodies overnight at 4°C. After washing three times with PBS, the cells were incubated with the corresponding secondary antibodies at 37°C for 1 h.

### Cytotoxicity assay

Briefly, the cytotoxicities of the test compounds in Vero and LLCPK cells were evaluated using the CCK-8 assay. Cells were incubated with different concentrations of the test compounds at 37°C for 24 h in a 5% CO_₂_ atmosphere. After incubation, the culture medium was removed, and the cells were washed three times with PBS before adding the CCK-8 solution. Finally, the cells were incubated at 37°C for 1 h, and the absorbance was measured at 450 nm using a microplate reader.

### Indirect immunofluorescence assay

Briefly, treated Vero cells were fixed with 4% paraformaldehyde for 10 min, permeabilized with Triton X-100 for 10 min, blocked with 5% bovine serum albumin (BSA) for 1 h, and incubated with a monoclonal antibody against PEDV N protein (1:500) overnight at 4°C. Thereafter, the cells were incubated with CoraLite 488 goat antimouse IgG secondary antibody at 37°C for 1 h. After three washes with PBS, the cells were observed using an inverted fluorescence microscope (Nikon, Tokyo, Japan).

### Quantitative real-time PCR (qRT-PCR)

Total RNA was extracted using RNAfast200 (Fastagen, Shanghai, China) and then reverse-transcribed into cDNA. Specific primers were designed based on the gene sequences listed in Table 1. GAPDH was used as a reference gene. CT values were determined using a real-time fluorescence quantitative PCR system (Bio-Rad, Hercules, CA, USA).

**TABLE 1:** qRT-PCR primers used in this study

Primer	Sequence (5′–3′)
CTSB-F	TTCGGGCGTCAGAACCTG
CTSB-R	ATCCTAGATTCACCGCGCTC
CTSL-F	CCAGTTTTACAGCACAGGCAT
CTSL-R	GCCATCCATGCCCCAAGTAT
PEDV-N-F	CTCCTACTTCACGTGCAAA
PEDV-N-R	AGCTCCACGACCCTGGTTAT
PEAV-N-F	CTGACTGTTGTTGAGGTTAC
PEAV-N-R	TCTGCCAAAGCTTGTTTAAC
PDCoV-N-F	CCCTTACCTTCTCTTACTCAATCAC
PDCoV-N-R	AGGTTTCTTCTGCTGTTTGGG
GAPDH-F	CCTTCCGTGTCCCTACTGCCAAC
GAPDH-R	GACGCCTGCTTCACCACCTTCT
IL-6-F	TACTGGCAGAAACAACCTG
IL-6-R	GTACTAATCTGCACAGCCTC
IL-8-F	AGTTTTCCTGCTTTCTGCAGCT
IL-8-R	TGGCATCGAAGTTCTGCACT
TNF-α-F	CCTACTGCACTTCGAGGTTATC
TNF-α-R	GCATACCCACTCTGCCATT

### Time-of-addition assay

To determine the exact stage of the viral replication cycle at which the test compound exerts its antiviral effects, samples were collected at different time points for analysis. Vero cells were pretreated with magnolol for 2 h and then infected with PEDV (MOI = 1) starting at 0 h and continuing for 2 h. At 0–2, 2–4, 4–6, 6–8, 8–10, and 10–12 h, the cells were washed three times with PBS or treated with 20 μM magnolol, followed by incubation in DMEM containing 10 μg/mL trypsin. PEDV-infected or uninfected Vero cells were incubated at 37°C for 24 h in a 5% CO_2_ atmosphere and then collected for Western blot analysis.

### Virus adsorption and internalization assay

For the adsorption assay, the cells were coincubated with PEDV and magnolol in 12-well plates at 4°C for 1 h. After three washes with PBS, the medium was replaced with a virus maintenance medium and incubated at 37°C. For the internalization assay, cells were incubated with PEDV (MOI = 0.1) at 4°C for 1 h, followed by washing with PBS to remove any unbound virus. Thereafter, the medium was replaced with 20 μM magnolol, and the cells were incubated at 37°C for 24 h.

### Western blotting

Briefly, cells were lysed in radioimmunoprecipitation assay buffer at 4°C for 30 min and centrifuged at 10,000 × *g* for 10 min at 4°C to collect the lysates. Thereafter, proteins were separated using SDS-PAGE and transferred to polyvinylidene difluoride membranes (0.45 μm, Merck Millipore) for detection, with GAPDH as a loading control. After blocking with 5% skim milk for 1 h, the membranes were washed three times with Tris-buffered saline containing 0.05% Tween-20 (TBST, 5 min each) and incubated with the appropriate primary antibodies overnight at 4°C. After washing with TBST for 5 min, the membranes were incubated with secondary antibodies at room temperature for 1 h. Finally, protein bands were visualized and analyzed using a dual-laser imaging system (Azure Biosystems, Dublin, CA, USA).

### Immunofluorescence confocal microscopy

IPEC cells were cultured in confocal dishes (Nest Biotechnology, Wuxi, China) and infected with PEDV (MOI = 50). After allowing the virus to adsorb at 4°C for 1 h, the cells were washed three times with PBS to remove any unbound viruses. Thereafter, the cells were incubated with virus maintenance medium or 20 μM magnolol at 37°C for 40 min, fixed in 4% paraformaldehyde for 15 min, and permeabilized with 0.3% Triton X-100 for 10 min. After blocking with 5% BSA at 37°C for 1 h, the cells were incubated with the appropriate primary antibodies overnight at 4°C. Finally, the cells were washed three times with PBS and incubated with the corresponding secondary antibodies at 37°C for 1 h.

### Endosomal acidification assay

LysoTracker Green (Beyotime Biotechnology) uses an acidophilic fluorescent probe to label and track acidic organelles in living cells. After seeding IPEC cells in confocal dishes, the cells were treated with PEDV (MOI = 0.1) at 4°C for 1 h. Baf-A1 and CQ were used as positive controls, and untreated PEDV-infected cells served as negative controls. After washing, the cells were incubated with magnolol at 37°C for 1 h, followed by incubation with 50 nM LysoTracker Green working solution at 37°C for 30 min. Finally, fluorescence was observed immediately using a confocal laser scanning microscope.

### Animal experiment

Twelve piglets (3 days old) were randomly divided into three groups (four piglets per group): negative control (uninfected), positive control (infected but untreated), and treatment (infected and treated). Piglets in the treatment group were orally administered 100 mg/kg magnolol every 24 h for three consecutive days, whereas those in the negative and positive control groups received an equivalent volume of DMEM.

During the experiment, body weights and rectal temperatures were monitored daily and compared with those of the control group. Clinical signs and survival rates were recorded during the study period. Rectal swabs were collected to determine viral RNA copy numbers. Dead or morbid piglets were immediately necropsied. All surviving piglets were euthanized and necropsied 9 dpi. Intestinal tissues from the duodenum, jejunum, ileum, and rectum were collected for histopathological and viral load analyses.

### Statistical analysis

All statistical analyses and calculations were performed using GraphPad Prism software (GraphPad Software, San Diego, CA, USA). Significant differences between groups were analyzed using analysis of variance and *t*-test. All experimental data are presented as mean ± standard deviation. Statistical significance was set at *P* < 0.05 (*P < 0.05, **P < 0.01, ***P < 0.001, and ****P < 0.0001).

## Data Availability

The data that support the findings of this study are available from the corresponding author upon reasonable request.

## SUPPLEMENTAL MATERIAL

Supplemental material jvi.00137-26-s0001.pdf Fig. S1 to S5.

ASM does not own the copyrights to Supplemental Material that may be linked to, or accessed through, an article. The authors have granted ASM a non-exclusive, world-wide license to publish the Supplemental Material files. Please contact the corresponding author directly for reuse.
