# Supplementary material for: Magnolol inhibits porcine epidemic diarrhea virus infection by suppressing cathepsin L expression in vitro and in vivo
Source: J Virol. 2026 Jun 25;100(7):e00137-26. doi: 10.1128/jvi.00137-26 (PMC13386832; doi:10.1128/jvi.00137-26)
Supplement: Supplemental material — Fig. S1 to S5. [file jvi.00137-26-s0001.pdf]

Supplementary Figures

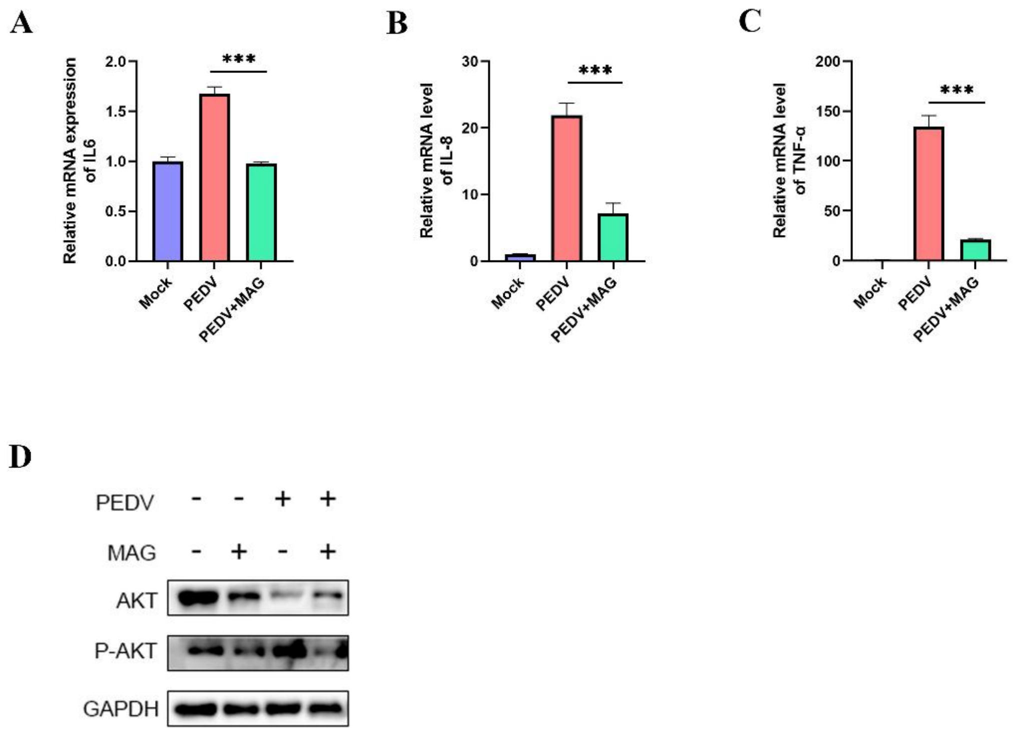

Figure S1. (A–C) Effects of magnolol on the mRNA expression levels of IL-6, IL-8, and TNF-α in PEDV-infected cells as determined by RT-qPCR. Significance compared with the infection group is indicated as *** ($p < 0.001$). (D) Western blot analysis of the effects of magnolol on AKT and p-AKT expression.

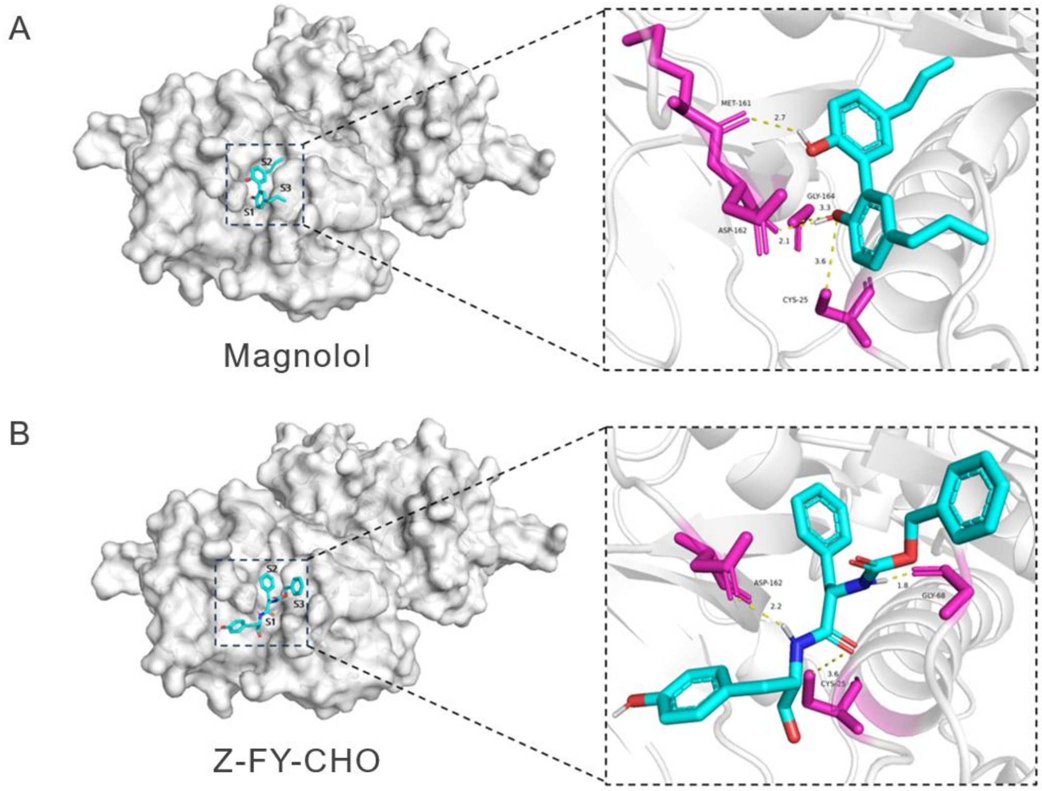

Figure S2. Molecular docking analysis of magnolol and Z-FY-CHO with CTSL. (A) Binding mode of magnolol (blue sticks) with CTSL (white surface). (B) Binding mode of Z-FY-CHO (blue sticks) with CTSL (white surface). Key interacting amino acid residues are shown as pink sticks, and hydrogen bonds are represented by yellow dashed lines.

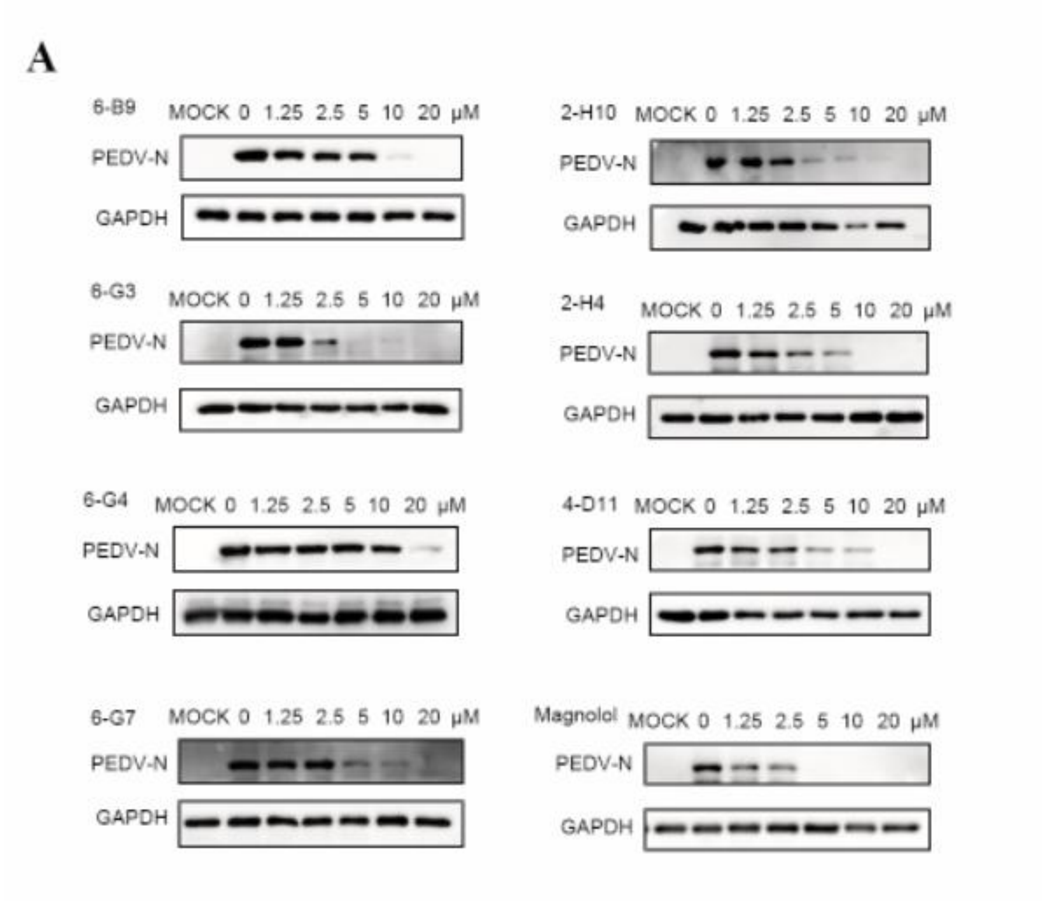

Figure S3. Effects of candidate compounds at different concentrations on PEDV N protein expression in IPEC cells. (A) Western blot identification.

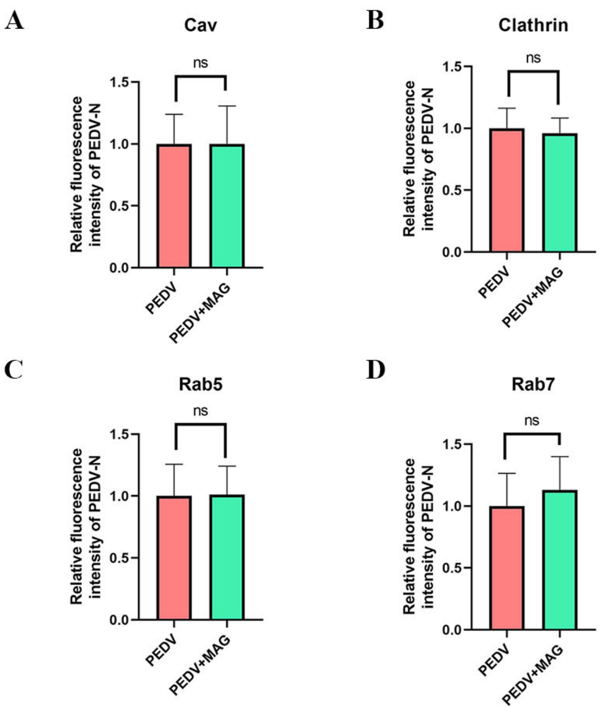

Figure S4. Changes in PEDV N protein fluorescence intensity in IPEC cells after magnolol treatment, as observed by confocal microscopy. Significance compared with the infection group is indicated as ns ($p > 0.05$).

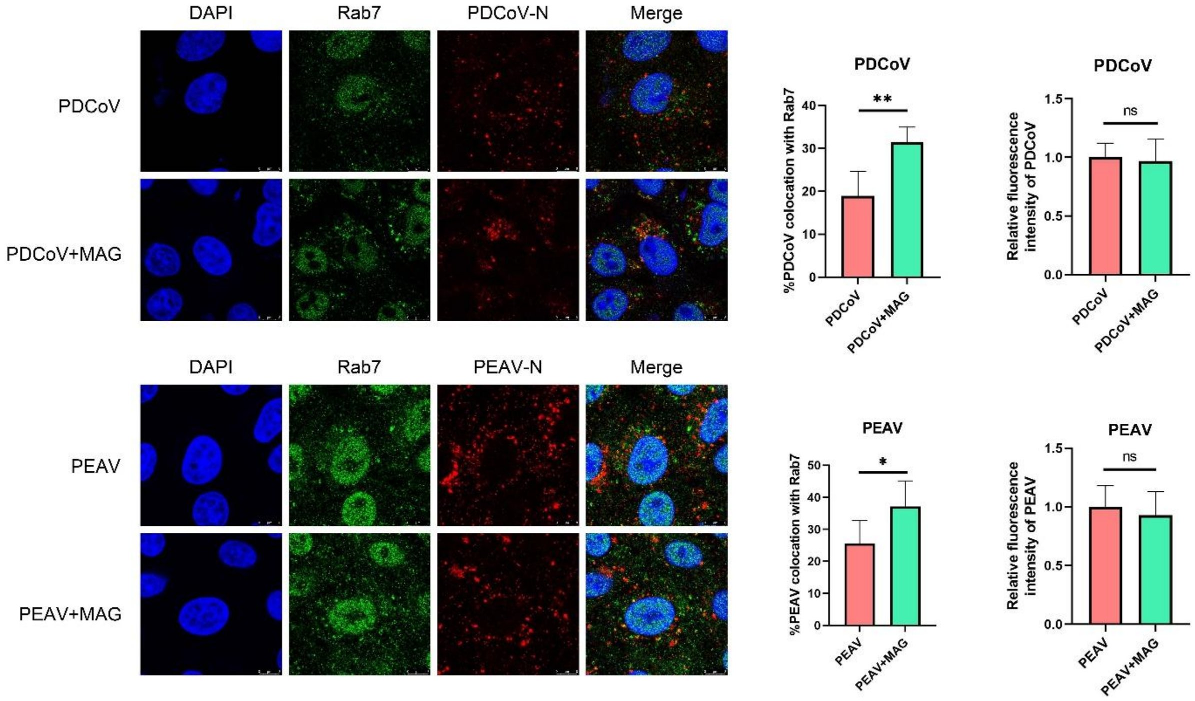

Figure S5. Changes in the colocalization of PEAV and PDCoV N proteins with Rab7 following magnolol treatment. Significance compared with the infection group is indicated as ns ($p > 0.05$), * ($p < 0.05$) and ** ($p < 0.01$).
